# Alternatives to antibiotics as growth promoters for use in swine production: a review

**DOI:** 10.1186/2049-1891-4-35

**Published:** 2013-09-14

**Authors:** Philip A Thacker

**Affiliations:** 1 Department of Animal and Poultry Science, University of Saskatchewan, 51 Campus Drive, Saskatoon, Saskatchewan S7N 5A8, Canada

**Keywords:** Antimicrobial peptides, Clay minerals, Egg yolk antibodies, Essential oils, Eucalyptus oil-medium chain fatty acids, Rare earth elements, Recombinant enzymes

## Abstract

In the past two decades, an intensive amount of research has been focused on the development of alternatives to antibiotics to maintain swine health and performance. The most widely researched alternatives include probiotics, prebiotics, acidifiers, plant extracts and neutraceuticals such as copper and zinc. Since these additives have been more than adequately covered in previous reviews, the focus of this review will be on less traditional alternatives. The potential of antimicrobial peptides, clay minerals, egg yolk antibodies, essential oils, eucalyptus oil-medium chain fatty acids, rare earth elements and recombinant enzymes are discussed. Based on a thorough review of the literature, it is evident that a long and growing list of compounds exist which have been tested for their ability to replace antibiotics as feed additives in diets fed to swine. Unfortunately, the vast majority of these compounds produce inconsistent results and rarely equal antibiotics in their effectiveness. Therefore, it would appear that research is still needed in this area and that the perfect alternative to antibiotics does not yet exist.

## Background

Antibiotics have played a major role in the growth and development of the swine industry for more than 50 years. Their efficiency in increasing growth rate, improving feed utilization and reducing mortality from clinical disease is well documented [1]. However, consumers are becoming increasingly concerned about drug residues in meat products [2]. In addition, it has been suggested that the continuous use of antibiotics may contribute to a reservoir of drug-resistant bacteria which may be capable of transferring their resistance to pathogenic bacteria in both animals and humans [3]. As a result, many countries have banned or are banning the inclusion of antibiotics in swine diets as a routine means of growth promotion.

In the past two decades, an intensive amount of research has been focused on the development of alternatives to antibiotics to maintain swine health and performance and many excellent reviews have already been published on this subject. The most widely researched alternatives include probiotics [4-6], prebiotics [4,7], enzymes [8-10], acidifiers [11-14], plant extracts [4,15,16] and neutraceuticals such as copper and zinc [17,18]. Since these additives have been more than adequately covered, the focus of this review will be on less traditional alternatives.

### Antimicrobial peptides

Antimicrobial peptides, as the name implies, are peptides with antimicrobial properties. They have been isolated and characterized from virtually all living organisms ranging from prokaryotes to humans [19]. They are important components of the host’s defense system and are effector molecules of innate immunity with direct antimicrobial and mediator function [20]. Most antimicrobial peptides contain between 30 and 60 amino acids and are polar molecules with spatically separated hydrophobic and charged regions. Antimicrobial peptides have been identified that have activity against Gram-positive and Gram-negative bacteria as well as against fungi and enveloped viruses [20].

More than 700 antimicrobial peptides are known to exist [20]. Bioscreening, cloning strategies and computer-based database searches have been used to identify antimicrobial peptides which have potential to be used as alternatives to antibiotics [20]. Once identified, it is possible to chemically synthesize most antimicrobial peptides but the high cost of this process precludes the production of peptides through this method for use as feed additives. However, several research groups have developed recombinant systems for expression of antimicrobial peptides.

Antimicrobial proteins produced by bacteria are called bacteriosins. These proteins have several characteristics that make them desirable alternatives to conventional antibiotics for use in swine production. Most importantly, bacteria have difficulty in developing resistance against these peptides [21]. Peptides have a narrow spectrum of activity so they can be used to target specific pathogenic bacteria without affecting the normal native flora. There is almost no risk of residues in meat because they are proteins and therefore will not be absorbed as an intact molecule. In addition, antimicrobial peptides can tolerate a wide range of pH and temperatures [22].

The antimicrobial activity of peptides is based on several mechanisms. In most cases, interactions between the peptide and the surface membranes of the target bacteria are thought to be responsible for their killing activity [20]. These interactions are proposed to lead to a loss of membrane function including breakdown of membrane potential, leakage of metabolites and ions, and alteration of membrane permeability [19]. These alterations in the bacterial membrane can result in cell lysis or, alternatively, can lead to the formation of transient pores and the transport of peptides inside the cell bringing them into contact with intracellular targets. Other mechanisms of antimicrobial activity include the inhibition of protein and RNA synthesis [20].

To date, the most prevalent use of antimicrobial peptides has been in the preservation of foods and few studies have been conducted using antimicrobial peptides with swine. One promising research area has been in the use of the antimicrobial peptide colicin. Colicins are a class of bacteriocin produced by and effective against *Escherichia coli* (*E. coli*) and closely related species. They have been shown to be effective against many pathogenic *E. coli* strains including those responsible for post-weaning diarrhea and edema disease in pigs [23,24].

A chemically synthesized antimicrobial peptide A3 has been shown to have beneficial effects on weanling pig performance, nutrient digestibility, intestinal morphology as well as intestinal and fecal microflora [25,26]. In addition, an antimicrobial peptide isolated from the intestine of the Rongchang pig improved performance but had no effect on diarrhea incidence in weanling pigs [27]. However, the antimicrobial peptide appeared to act synergistically with zinc as the two additives in combination were superior to either additive fed separately.

The results of a feeding trial in which the antimicrobial peptide cecropin, originally isolated from the silkworm *Hyalophora cecropia*, was fed to weanling pigs challenged with enterotoxigenic *E. coli K88* are shown in Table 1. Use of the antimicrobial peptide cecropin resulted in similar performance to pigs fed a combination of antibiotics [21]. The improvement in performance appeared to be related to improvements in nutrient digestibility and intestinal morphology. Cecropin treatment decreased total aerobes while increasing total anaerobes in the ileum compared with the control (Table 2). Cecropin also increased the numbers of beneficial lactobacillus in the cecum. Cecropin increased serum IgA and IgG and the inflammatory cytokines interleukin-1β and interleukin 6 indicating that cecropin activates both systemic and local immune systems in response to *E. coli* challenge.

**Table 1:** **Effects of antibiotics or an antimicrobial peptide cecropin on the performance of four week old weaned pigs after challenge with *E. coli *as well as nutrient digestibility before challenge**

**Items**	**Control**	**Antibiotics**^**1**^	**Cecropin**	**SEM**	***P*-value**
**Performance (day 13-19)**					
Weight gain, g/d	312^a^	367^b^	358^b^	6.4	<0.01
Feed intake, g/d	566	597	592	9.8	0.08
Feed efficiency	0.55^a^	0.62^b^	0.61^b^	0.01	<0.01
Diarrhea incidence, %	37.50	17.86	19.64		
**Nutrient digestibility**					
Nitrogen retention, g/d	10.1^a^	11.5^b^	10.7^ab^	0.38	0.04
Nitrogen digestibility, %	73.2	76.9	75.0	1.20	0.17
Energy retention, MJ/kg/d)	2.5^a^	3.0^b^	2.8^ab^	0.13	0.04
Energy digestibility, %	84.6	88.2	86.4	1.71	0.14

Wu et al. [21].

^1^Kitasamycin and colistin sulfate.

^a,b^Within row, means followed by same or no letter do not differ (*P*>0.05).

**Table 2:** **Effects of antibiotics or the antimicrobial peptide cecropin on intestinal morphology and intestinal microflora of four week old weaned pigs after challenge with *E. coli***

**Items**	**Control**	**Antibiotic**^**1**^	**Cecropin**	**SEM**	***P*-value**
**Intestinal Morphology**					
Duodenum					
Villus height, μm	418	439	431	10.7	0.53
Crypt depth, μm	233	227	232	5.3	0.41
Villus height to crypt depth ratio	1.83	1.96	1.89	0.24	0.18
Jejunum					
Villus height, μm	401	448	420	18.4	0.37
Crypt depth, μm	212^b^	233^a^	220^b^	6.8	0.04
Villus height to crypt depth ratio	1.89^b^	1.97^a^	1.91^ab^	0.01	0.03
Ileum					
Villus height, μm	357^b^	396^a^	384^a^	12.4	0.04
Crypt depth, μm	211	217	213	5.6	0.37
Villus height to crypt depth ratio	1.74^b^	1.85^a^	1.82^ab^	0.04	0.04
**Intestinal Microflora (log**_**10 **_**CFU/g of digesta)**					
Ileum					
*E. coli*	4.37	4.14	4.25	0.18	0.85
*Lactobacillus*	9.38	10.00	9.62	0.20	0.42
Total aerobes	6.69^a^	6.60^ab^	6.43^b^	0.08	0.04
Total anaerobes	9.36^b^	9.87^ab^	10.12^a^	0.23	0.03
Cecum					
*E. coli*	3.37^a^	3.09^b^	3.22^ab^	0.12	0.04
*Lactobacillus*	8.89^b^	9.47^a^	9.23^a^	0.14	0.03
Total aerobes	3.88	3.77	3.49	0.44	0.63
Total anaerobes	8.79	9.37	9.26	0.28	0.38

Wu et al. [21].

^1^Kitasamycin and colistin sulfate.

^a,b^Within row, means followed by same or no letter do not differ (*P*>0.05).

Although there is little research on these compounds, the use of antimicrobial peptides appears to have considerable potential as a replacement for antibiotics in rations fed to swine. A commercial entity (Beijing Longkefangzhou Biological Engineering Technology Company, Beijing, China) has started to market cecropin for use in swine rations in China.

### Clay minerals

Clay minerals are formed by a net of stratified tetrahedral and octahedral layers [2]. They contain molecules of silicon, aluminum and oxygen. The natural extracted clays (bentonites, zeolite, kaolin) are a mixture of various clays differing in chemical composition. The best known are montmorillonite, smectite, illite, kaolinite, biotic and clinoptilolite [2].

Clays added to the diet can bind and immobilize toxic materials in the gastrointestinal tract of animals and thereby reduce their biological availability and toxicity [2]. Clay minerals can bind aflatoxins, plant metabolites, heavy metals, and toxins. The extent of adsorption is determined by the chemistry of the clay minerals, exchangeable ions, surface properties and the fine structure of the clay particles [2]. An important role is played by pH, dosage and exposure time. As a result of their binding properties, clays have been widely used in swine diets to improve pig performance when diets containing mycotoxins are fed [28,29].

Clays have also been shown to prevent diarrhea in weaned pigs [2,30,31]. Based on this fact, several research groups have attempted to determine whether or not the inclusion of various clays in swine diets can improve pig performance. The results have been inconclusive with some trials demonstrating positive results particularly for younger pigs [30], but the vast majority of the experiments have failed to show improvements [32-35]. It would appear that clay minerals are not viable alternatives to antibiotics as growth promoters.

### Egg yolk antibodies

One technique that appears to have considerable potential as an alternative to antibiotics for growth promotion in the presence of disease causing organisms is the use of egg yolk antibodies generally referred to as IgY [36]. In order to produce these antibodies, laying hens are injected with organisms that cause specific diseases in swine. The injection of these antigens induces an immune response in the hen which results in the production of antibodies. These antibodies are typically deposited in the egg yolk. Booster immunizations are given to ensure continued transfer of antibodies from the hen to the egg yolk. These antibodies are then extracted from the egg yolk and processed. Antibodies can be administered in the feed in several forms including whole egg powder, whole yolk powder, water-soluble fraction powder or purified IgY [37]. Details concerning IgY production including choice of adjuvant, route of immunization, dose, immunization frequency and techniques for IgY extraction from the yolk have been reviewed by Chalghoumi et al. [37] and Kovacs-Nolan and Mine [38].

Compared with the use of mammals such as rabbits or sheep for antibody production, the immunization of chickens for antibody production is an attractive approach. Chicken housing is inexpensive, egg collection is non-invasive, the IgY antibodies are concentrated in egg yolk and isolation is fast and simple. In addition, chicken immunnoglobin does not react with mammalian IgG or IgM and also it does not activate mammalian complement factors [38]. Finally, the use of IgY elicits no undesirable side effects, disease resistance or toxic residues [36].

IgY antibodies have been tested against a number of enteric pathogens in swine including *E. coli, Salmonella* and *Rotaviru*s with varying degrees of success [39-43]. Table 3 shows the results of an experiment where the performance of pigs fed egg yolk antibodies was compared with that of pigs fed diets supplemented with zinc oxide, fumaric acid or antibiotics. All four feed additives successfully increased pig performance compared with unsupplemented pigs with significant reductions observed in scour score and piglet mortality. In this experiment, egg yolk antibody was equal to antibiotics in enhancing pig performance.

**Table 3:** Effect of egg yolk antibody, zinc oxide, fumaric acid and antibiotic on the performance and intestinal morphology of 10 to 24 day old pigs fed diets based on pea protein concentrate

**Items**	**Control**	**Egg yolk antibody**	**Zinc oxide**	**Fumaric acid**	**Carbadox**	**SEM**
Weight gain, g/d	100.9	151.2	158.9	155.4	152.6	16.6
Feed intake, g/d	141.0	208.1	214.7	211.6	222.4	15.3
Feed conversion	1.39	1.38	1.35	1.36	1.45	0.04
Scour score	2.7	1.3	1.4	1.3	1.1	-
Mortality, %	40.0	6.6	13.3	6.6	13.3	-
Villus height, m	355	564	488	573	570	20.0
Crypt depth, m	204	183	190	207	204	10.1
Villous height:crypt depth	1.7	3.1	2.6	2.8	2.8	0.11

Owusu-Asiedu et al. [41].

Unfortunately, there are several reports where egg yolk antibody failed to improve pig performance [42,44]. The most likely explanation for the failure of egg yolk antibody to improve performance is that the antibody failed to survive passage through the gastrointestinal tract [45]. It appears that the IgY molecule is less stable than the IgG molecule due to its higher molecular weight, lower percentage of β-sheet structure and reduced flexibility [45]. It has been reported that the activity of IgY was decreased at pH 3.5 or lower and almost completely lost activity with irreversible change at pH 3 [37]. In addition, IgY is fairly sensitive to pepsin digestion [45]. Therefore, a recent avenue of research has been to use microencapsulation techniques to protect IgY from gastric inactivation [46,47].

Table 4 shows the results of an experiment where chitosan-alginate microcapsules were used for oral delivery of egg yolk immunoglobulin in weaned pigs challenged with enterotoxigenic *E. coli* C83903 [46]. The percentage of pigs with diarrhea 24 h after treatment and the diarrhea score were improved in pigs receiving encapsulated IgY compared with non-encapsulated IgY. In addition, weight gain over the three day period was significantly higher in pigs receiving encapsulated IgY compared with non-encapsulated IgY. Both encapsulated and non-encapsulated IgY treatments were numerically superior to an aureomycin treated group.

**Table 4:** **Effect of encapuslation of IgY on performance and the incidence of diarrhea in pigs challenged with *E. coli***

	**Percentage of pigs with diarrhea after specific times (Fecal score in brackets)**^**1**^					
**Items**	**9 h**	**24 h**	**48 h**	**72 h**	**Weight gain (g/d)**	**Recovery rate (%)**
Negative control, unchallenged	0% (0.5)	0% (0.0)	0% (0.4)	0% (0.0)	116.6^a^	-
Positive control	75% (2.5)	75% (2.5)	75% (2.0)	75% (2.0)	13.5^d^	0%
Non-encapsulated IgY	100% (2.0)	75% (1.3)	25% (1.0)	0% (0.0)	78.1^b^	100%
Microencapsulated IgY	75% (2.0)	0% (0.0)	0% (0.0)	0% (0.0)	110.4^a^	100%
Aureomycin	100% (2.0)	50% (2.0)	75% (1.5)	50% (1.5)	54.1^c^	50%

Li et al. [46].

^1^Fecal score is the mean fecal consistency score where 0 = normal, 1 = soft feces, 2 = mild diarrhea, 3 = severe diarrhea.

^a,b,c,d^Within column, means followed by same or no letter do not differ (*P*>0.05).

The mechanism through which IgY counteracts pathogen activity has not been determined. However, several mechanisms were proposed by Xu et al. [36] including agglutination of bacteria, inhibition of adhesion, opsonization followed by phagocytosis and toxin neutralization. Further research is necessary to determine the exact mechanism for the growth promoting activity of IgY.

### Essential oils

Essential oils are aromatic oily liquids obtained from plant material and usually have the characteristic odor or flavor of the plant from which they are obtained [48]. They are typically mixtures of secondary plant metabolites and may contain phenolic compounds (i.e. thymol, carvacrol and eugenol), terpenes (i.e. citric and pinapple extracts), alkaloids (capsaicine), lectins, aldehydes (i.e. cinnamaldehyde), polypeptides or polyacetylenes [49]. They can be extracted from plants with organic solvents or steam distillation [49]. An estimated 3000 essential oils are known to exist but cinnamaldehyde, carvacrol, eugenol and thymol have received the most interest for use in swine production.

Interest in the use of essential oils as a potential replacement for antibiotics in swine rations has been generated as a result of *in vitro* studies showing that essential oils have antimicrobial activity against microflora commonly present in the pig gut [50]. The exact mode of action of essential oils has not been established but the activity may be related to changes in lipid solubility at the surface of the bacteria [48]. The hydrophobic constituents of essential oils allow them to disintegrate the outer membrane of *E. coli* and *Salmonella* and thus inactivate these pathogens [48]. This would result in a shift in the microbial ecology in favor of lactic acid producing bacteria and reducing the number of pathogenic bacteria [50]. Essential oils containing phenolic compounds tend to have greater antimicrobial activity than oils containing other compounds [51].

Based on the fact that essential oils appear to control pathogenic bacteria, several research groups have attempted to determine whether or not the inclusion of essential oils in swine diets can improve pig performance [52]. The results have been inconclusive with some trials demonstrating positive results [53-55] while others have reported no beneficial effects [56,57]. The most compelling evidence for including essential oils in diets fed to swine can be obtained from the results of Li et al. [55]. This trial compared the performance of pigs fed an unsupplemented control diet with that of pigs fed a diet supplemented with antibiotics or a combination of thymol and cinnamaldehyde (Table 5). Weight gain, feed conversion and fecal consistency of pigs fed essential oils was essentially equal to that of pigs fed antibiotics. The improved performance appeared to be mediated by improvements in dry matter and protein digestibility arising from improvements in intestinal morphology. In addition, total antioxidant capacity and levels of the cytokines interleukin-6 and tumor necrosis factor-α were altered by inclusion of essential oils (Table 6).

**Table 5:** Effect of essential oils on weanling pig performance, nutrient digestibility and fecal consistency

**Items**	**Control**	**Antibiotic**^**1**^	**Essential oil**	**SEM**
**Performance**				
Weight gain, g/d	442^a^	505^b^	493^b^	15
Feed intake, g/d	783	846	789	24
Feed conversion	1.79	1.67	1.62	0.06
Fecal consistency	1.53^a^	1.22^b^	1.30^b^	0.06
**Nutrient digestibility**				
Dry matter	84.33^a^	87.03^b^	86.92^b^	0.65
Crude protein	76.51^a^	83.53^b^	81.34^b^	1.25

Li et al. [55].

^1^Chlortetracycline, colistin sulfate and kitasamycin.

^a,b^ Within row, means followed by same or no letter do not differ (*P*>0.05).

**Table 6:** Effect of essential oils on intestinal morphology, antioxidant capacity, and cytokine levels in weanling pigs

**Items**	**Control**	**Antibiotic**^**1**^	**Essential oil**	**SEM**
Villus height, μm	466	509	535	24
Crypt depth, μm	164	156	162	8
Villus height:crypt depth	2.96^a^	3.41^b^	3.38^b^	0.09
Total antioxidant capacity, U/mL	10.46^a^	11.97^ab^	12.37^b^	0.52
Interleukin-6, ng/L	44.21^a^	40.39^a^	27.40^b^	2.76
Tumor necrosis factor-α, ng/L	208^a^	237^ab^	260^b^	13

Li et al. [55].

^1^Chlortetracycline, colistin sulfate and kitasamycin.

^a,b^Within row, means followed by same or no letter do not differ (*P*>0.05).

The reason for the variability in results when essential oils are fed is likely due to differences in the type of essential oils used and the dose provided [55]. As noted previously, oils containing phenolic compounds tend to have greater antimicrobial activity than those based on other compounds. In addition, if the dose used is too high, the strong smell can reduce feed intake and thereby limit pig performance [48]. Another important consideration is the stability of essential oils during pelleting. Maenner et al. [54] reported considerable loss of activity of essential oils when a pelleting temperature of 58°C was applied.

### Eucalyptus oil-medium chain fatty acids

Eucalyptus oil is obtained from the leaves of the eucalyptus, a tree which belongs to the plant family *Myrtaceae* and is cultivated worldwide. In humans, eucalyptus oil has been shown to have antibacterial effects on pathogenic bacteria in the respiratory tract [58]. Eucalyptus oil has also been shown to stimulate the immune system by affecting the phagocytic ability of monocyte-derived macrophages [59]. In poultry, dietary inclusion of eucalyptus has been shown to improve production performance and stimulate the immunity of commercial laying hens [60].

Medium-chain fatty acids have been suggested as an alternative feed additive to antibiotics for piglets [61-63]. Medium chain fatty acids have been shown to have antimicrobial activity against *Salmonella*[64] and *E. coli*[61]. Hong et al. [63] reported that feeding a blend of caprylic and caproic acids improved performance and nutrient digestibility in 3 and 4 week old weaned pigs during the first two weeks following weaning.

Micro-encapsulation of medium chain fatty acids is a process in which medium chain fatty acids are nano-micronized to extremely small particles and then encapsulated. Han et al. [65] tested a product where eucalyptus extract was mixed with caprylic and carpric acids and encapsulated with palm oil in comparison with antibiotics or zinc oxide (Table 7). The performance of pigs fed the eucalyptus-medium chain fatty acid blend was essentially equal to that of antibiotics or zinc oxide. The performance enhancing effects of the blend appeared to be mediated through improvements in nutrient digestibility (Table 8). The process used to produce the micro-encapsualted eucalyptus-medium chain fatty acid blend has been patented by the Korean Intellectual Property Office under patent number 10-2009-0025329.

**Table 7:** Effects of antibiotics, zinc oxide, and eucalyptus-medium chain fatty acids (MCFA) on nursery pig performance

**Items**	**Control**	**Antibiotics**^**1**^	**ZnO ( 1,500 ppm)**	**ZnO (2,500 ppm)**	**Eucalyptus-MCFA**	**SEM**	***P***
Weight gain, g/d	243^a^	315^b^	298^b^	308^b^	310^b^	13.6	<0.01
Feed intake, g/d	361^a^	431^b^	426^b^	429^b^	448^b^	18.1	<0.01
Feed conversion	1.53	1.41	1.44	1.41	1.46	0.05	0.35

Han et al. [65].

^1^Tiamulin and lincomycin.

^a,b^Within row, means followed by same or no letter do not differ (*P*>0.05).

**Table 8:** Effects of antibiotics, zinc oxide, and eucalyptus-medium chain fatty acids (MCFA) on nutrient digestibility for weaned pigs

**Items**	**Antibiotics**^**1**^	**ZnO (1,500 ppm)**	**ZnO (2,500 ppm)**	**Eucalyptus-MCFA**	**SEM**	***P***
Dry matter	91.74^a^	90.58^b^	90.44^b^	92.17^a^	0.26	< 0.01
Crude protein	74.18^a^	72.01^a^	71.23^a^	78.93^b^	1.13	< 0.01
Calcium	56.31^a^	48.26^b^	46.75^b^	65.93^c^	1.56	<0.01
Phosphorus	54.48^a^	38.25^b^	42.77^b^	66.10^b^	2.01	<0.01
Energy	82.92^a^	81.60^b^	81.00^b^	86.00^c^	0.61	< 0.01
Lysine	79.13^a^	80.25^b^	78.25^a^	83.80^b^	0.88	< 0.01
Methionine	83.94^a^	80.95^b^	80.78^b^	84.23^a^	0.63	<0.01
Threonine	73.56^b^	73.57^b^	73.43^b^	79.40^a^	1.40	0.02

Han et al. [65].

^1^Tiamulin and lincomycin.

^a,b^Within row, means followed by same or no letter do not differ (*P*>0.05).

### Rare earth elements

Rare earth elements comprise the elements scandium, yttrium, lanthanum and the 14 chemical elements following lanthanum in the periodic table called lanthanoids [66]. The application of rare earth elements as feed additives for livestock has been practiced in China for decades [66]. There are many articles in the Chinese literature concerning the performance enhancing effects of rare earth elements for swine [67,68] and many more have been reviewed by Rambeck and Wehr [69] and Redling [66]. In the Chinese literature, body weight gain was shown to be improved by 5 to 23% and feed conversion between 4 and 19% under the influence of rare earth elements.

Research concerning the effect of rare earth elements on swine performance have been published in the Western literature since about the year 2000 with some reports indicating significant improvements in pig performance [70,71] while others have observed no change [72]. Table 9 shows the results of a recent trial in which the performance of weaned pigs fed a lanthanum-yeast mixture was similar to that of pigs fed diets supplemented with antibiotics or zinc oxide [73].

**Table 9:** Effects of zinc oxide, antibiotic, or lanthanum-yeast on the performance of weanling pigs (day 0 to 28)

**Items**	**Control**	**Antibiotic**^**1**^	**Zinc (1,500 ppm)**	**Zinc (2,500 ppm)**	**Lanthanum-yeast**	**SEM**	***P *Values**
Weight gain, g/d	302^b^	353^a^	352^a^	369^a^	359^a^	14.0	0.02
Feed intake, g/d	467^b^	518^ab^	530^ab^	558^a^	501^ab^	22.6	0.10
Feed conversion	1.55^a^	1.47^ab^	1.50^ab^	1.52^ab^	1.41^b^	0.04	0.31

Han and Thacker [73].

^1^Tiamulin and chlortetraccycline.

^a,b^ Within row, means followed by same or no letter do not differ (*P*>0.05).

The products commonly used as feed additives for swine are typically mixtures of rare earth elements mainly containing lanthanum, cerium and praseodymium [73]. Both inorganic and organic rare earth compounds have been used as feed additives but it is believed that best results are obtained with organic compounds [66].

Several mechanisms have been proposed for the growth promoting effects of rare earth elements. It has been suggested that rare earth elements may promote growth by influencing the development of undesirable bacterial species within the gastrointestinal tract. For example, lanthanum has been shown to bind to the surface of bacteria [69]. This reduces the surface charge and retards electrophoretic migration. When the surface charge is completely neutralized, flocculation occurs. In addition, bacterial respiration has been shown to be strongly inhibited by lanthanides [69].

Another explanation for the growth promoting effects of rare earth elements is due to improvements in nutrient digestibility and availability as was observed by Han and Thacker [73]; Table 10]. It has been suggested that rare earth elements may influence the permeability of the intestines thereby enhancing the absorption of different nutrients [66]. Enhanced secretion of digestive fluids and increased gastrointestinal motility have also been proposed as explanations for the enhanced digestibility of nutrients following dietary inclusion of rare earth elements [66].

**Table 10:** Effects of antibiotics, zinc oxide or lanthanum-yeast on nutrient digestibility

**Items**	**Antibiotics**^**1**^	**Zinc(1,500 ppm)**	**Zinc (2,500 ppm)**	**Lanthanum-yeast**	**SEM**	***P*-value**
Dry matter	95.19^a^	93.83^b^	93.98^b^	95.46^a^	0.30	<0.01
Crude protein	74.51^ab^	71.55^b^	72.33^b^	78.34^a^	1.38	0.01
Calcium	56.59^b^	46.98^c^	48.50^c^	65.10^a^	1.69	<0.01
Phosphorus	54.87^b^	43.07^c^	38.52^c^	66.11^a^	2.09	<0.01
Energy	83.51^b^	81.42^b^	81.33^b^	86.89^a^	0.80	<0.01
Lysine	81.45^b^	79.42^b^	80.32^b^	85.15^a^	0.95	<0.01
Methionine	83.49^b^	83.67^b^	86.76^a^	87.32^a^	0.79	<0.01
Phenylalanine	74.21^b^	73.75^b^	75.41^ab^	78.96^a^	1.32	0.05
Threonine	76.19^b^	75.13^b^	75.28^b^	81.19^a^	1.58	0.04

Han and Thacker [73].

^1^Tiamulin and chlortetraccycline.

^a,b,c^Within row, means followed by same or no letter do not differ (*P*>0.05).

Rare earth elements have several properties that make them attractive alternatives to antibiotics. Generally, absorption of orally applied rare earths is low with more than 95% being recovered in the feces of animals [66]. As a result, the chances of residues being present in meat are low with studies reporting no higher levels of rare earth elements in the muscle tissue of supplemented animals than those fed commercial diets [66]. In addition, there have been no reports of the development of bacterial resistance in treated animals [66].

### Recombinant enzymes

Enzymes are biologically active proteins that break specific chemical bonds to release nutrients for further digestion and absorption. They accelerate chemical reactions in the body which would otherwise proceed very slowly or not at all [74]. Enzymes used in the feed industry are commonly produced by bacteria (i.e. *Bacillus subtilis*), fungus (i.e. *Trichoderma reesei*, *Aspergillus niger*) or yeast (*Saccharomyces cerevisiae*).

The supplementation of swine diets with exogenous enzymes to enhance performance is not a new concept and research articles in this field date back to the 1950’s [10]. The most common reasons for enzyme supplementation include degrading feed components resistant to endogenous enzymes (i.e. β-glucanase, xylanase, mannanase, pectinase and galactosidase), inactivating antinutritional factors (i.e. phytase) and supplementing endogenous enzymes that may be present in insufficient amounts (i.e. proteases, lipases and amylases). This review will focus on the use of enzymes to degrade feed components resistant to endogenous enzymes.

The cell walls of cereal grains, legumes and oilseed meals are comprised of complex carbohydrates commonly referred to as non-starch polysaccharides [75]. Non-starch polysaccharides consist of a wide range of polymers which include cellulose, hemicellulose, pectins, β-glucans, α-galctosides (raffinose, stachnyose and verbascose) and xylans [8]. These non-starch polysaccharides reduce the nutritional value of feed ingredients in a number of ways [74]. Firstly, they are indigestible by mammalian enzymes and therefore dilute the energy and nutrient content of the feed. Secondly, non-starch polysaccharides exhibit a so called "cage effect" whereby normally highly digestible nutrients such as starch, fat and protein are entrapped in a coating of non-starch polysaccharides preventing access of the endogenous enzymes to these substrates [76]. In addition, certain non-starch polysaccharides may increase intestinal viscosity. It has also been suggested that non-starch polysaccharides allow microbial populations to assimilate a greater proportion of the nutrients contained in the feed into their own system thereby reducing the availability of these nutrients to the host [8].

Carbohydrases include all enzymes that catalyze a reduction in the molecular weight of polymeric carbohydrate but more than 80% of the global carbohydrase market is accounted for by xylanase and β-glucanase [10]. Other commercially available carbohydrases include α-amylase, β-mannanase, α-galactosidase and pectinase. These carbohydrases have widespread application in the poultry industry but are used less commonly in feeds for swine.

The effect of carbohydrase supplementation on the performance of pigs is inconsistent. There are reports of positive responses to carbohydrase supplementation [77,78], whereas others have reported no improvement in weight gain in response to enzymes [79-81]. Where positive effects on performance are observed, they are commonly associated with increases in nutrient digestibility likely as a result of increased accessibility of endogenous enzymes to nutrients as a result of inhibition of the "cage effect" as well as hydrolysis or partial hydrolysis of the non-starch polysaccharide. There also seems to be an influence on th e composition of the microflora in the digestive tract [76]. Hydrolysis of non-starch polysaccharides results in increased sugar release in the large and small intestine and thereby stimulates the growth of lactobacilli which produce lactic acid. Increased proportions of lactic acid promote gut health by suppressing the growth of coliforms such as pathogenic *E. coli.*

Based on a review of the literature, it is clear that the response of pigs to supplementation with carbohydrases is less consistent than has been observed with poultry. The question is why? What differences are there in the physiology of the pig and the chicken that might account for the differences in the magnitude of the results obtained. One clear difference is the pH in the gut. In the pig, the duration that feed is exposed to a low pH is significantly longer than in the chicken [82]. Therefore, it is possible that exposure to the low pH in the stomach of the pig is either partially or totally denaturing the enzyme accounting for the lower magnitude of responses obtained when carbohydrases are fed to pigs compared with poultry.

Many of the enzyme preparations used in the past were unsuitable for use in the harsh environment of the pig’s gastrointestinal tract. The pH in the stomach of the pig is usually between 2 and 3.5 and substantial reductions in β-glucanase [82] and xylanase [83] activity were reported when ten commercially available enzyme products were exposed *in vitro* to a pH of 2.5 or 3.5 for 30 min.

The application of genetic engineering in the process of enzyme production allows the development of enzymes targeted for specific purposes [84-86]. Recently, several carbohydrases have been developed by molecular directed evolution which have considerable potential for animal feed application [84-86]. Enzymes have been developed which are active over a broad pH range, exhibit thermostability, are resistant to pepsin and trypsin, and viable under simulated gastric conditions.

Inclusion of a recombinant β-mannanase in corn soybean meal diets fed to growing pigs increased weight gain by 16.1% and feed efficiency by 17.7% compared with an unsupplemented diet (Table 11). The magnitude of the improvement was notably greater than previous experiments using β-mannanase produced by traditional fermentation techniques. For example, Pettey et al. [87] reported that weight gain was only increased 3.4% and feed efficiency 3.9% in their experiment in which growing–finishing pigs were fed diets supplemented with β-mannanase.

**Table 11:** Comparison of the effects of a β-mannase produced using normal fermentation technology with that of a recombinant β-mannase on the performance of growing-finishing pigs

**Items**	**Control**	**β-mannase**	**% Improvement**
**Traditional fermentation**^**1**^			
Weight gain, g/d	0.84	0.87	3.4
Feed intake, g/d	2.50	2.48	-
Feed efficiency	0.337	0.351	3.9
**Recombinant technology**^**2**^			
Weight gain, g/d	0.66	0.79	16.4
Feed intake, g/d	1.66	1.61	3.0
Feed efficiency	0.404	0.491	17.7

^1^Pettey et al. [87].

^2^Lv et al. [85].

Enzymes added to feed are broken down in the digestive tract in the same way as other proteins [74]. Therefore, there are not any issues with residues and it is not necessary to observe any withdrawal periods before animals fed enzymes can be slaughtered [74]. For this reason, the amount of enzyme required is very small compared with the amount of substrate and therefore only small quantities are needed when using enzymes in ration formulation.

### Miscellaneous compounds

Many additional compounds have been tested for their potential to replace antibiotics as growth promoters for use in swine production. They are too numerous to be able to go into much detail regarding their effectiveness. Some of the more promising include spray-dried porcine plasma [88,89], yeast culture [90-92], bacteriophages [93], lysozyme [94], bovine colostrum [95], lactoferrin [96-98], conjugated fatty acids [99,100], chito-oligosaccarides [101,102] and seaweed extract [103].

## Conclusions

Clearly, a long and growing list of compounds exist which have been tested for their ability to replace antibiotics as feed additives to maintain swine health and performance. Unfortunately, the vast majority of these compounds produce inconsistent results and rarely equal antibiotics in their effectiveness. Therefore, it would appear that research is still needed in this area and that the perfect alternative does not exist as yet.

## Competing interests

The author declares they have no competing interests.
